# War Effects on Sickness

**Published:** 1915-08-28

**Authors:** 


### The Leicester Saturday Hospital Society.

### WAR EFFECTS ON SICKNESS.

Many interesting features marked the report of the above society presented to the half-yearly meeting of the contributors on Saturday. First and foremost was the expressed willingness and ready acquiescence of the members in supporting a previous decision to place the Desford Convalescent Home for men at the disposal of the military authorities as an auxiliary hospital. The committee had invested £2,500 of the society's funds in the War Loan, the report continued, an action which was approved by the members with equal readiness. In order to provide for male members of the society needing convalescent benefits the committee had adopted an expedient, which had been a source of considerable trouble and difficulty wherever it had been tried, of using the Swithland Convalescent Home for female members as a joint home for both sexes. This decision had not been without anxiety to the committee, but as a temporary measure they felt justified in taking the course they had done. In this connection acknowledgment was paid to the services of Miss Braye, the matron, and to Miss Havers, in the administration of the Desford Auxiliary Hospital, as well as to the medical officers of the homes. The report emphasised the gratifying fact that the state of local trade was in a flourishing condition, and consequently, although many of the members of the society had responded to the call for military service, it was pleasing that no ground had been lost, as upwards of 500 workpeople had been added to membership of the society during the half-year. Owing to the general decrease in sickness, the demands on the homes had been considerably less, 482 members, divided equally between men and women, having been admitted to the homes, a decrease of 119 on the figures for the corresponding period of last year. In addition, 196 military patients had been admitted to the Desford Auxiliary Hospital. The expenditure of the society had been £2,464, against an income of £2,124, and as £1,284 had been received from the Government in respect of military patients, there was a good prospect of the finances being in a satisfactory condition at the end of the year, notwithstanding the higher cost of all purchasable commodities. The regret of the members having been expressed at the continued ill-health of Sir Edward Wood, the president, the report was adopted.

